# Construction of the ETECFinder database for the characterization of enterotoxigenic *Escherichia coli* (ETEC) and revision of the VirulenceFinder web tool at the CGE website

**DOI:** 10.1128/jcm.00570-23

**Published:** 2024-04-24

**Authors:** Flemming Scheutz, Camilla Hald Nielsen, Astrid von Mentzer

**Affiliations:** 1 The International Escherichia and Klebsiella Centre, Statens Serum Institut, Copenhagen, Denmark; 2 Department of Bacteria, Parasites and Fungi, Statens Serum Institut, Copenhagen, Denmark; 3 Department of Microbiology and Immunology, Institute of Biomedicine, Sahlgrenska Academy, University of Gothenburg, Gothenburg, Sweden; Universität Münster, Münster, Germany

**Keywords:** enterotoxigenic *E. coli*, WGS tool, curated database, CGE website, ETEC virulence genes

## Abstract

The identification of pathogens is essential for effective surveillance and outbreak detection, which lately has been facilitated by the decreasing cost of whole-genome sequencing (WGS). However, extracting relevant virulence genes from WGS data remains a challenge. In this study, we developed a web-based tool to predict virulence-associated genes in enterotoxigenic *Escherichia coli* (ETEC), which is a major concern for human and animal health. The database includes genes encoding the heat-labile toxin (LT) (*eltA* and *eltB*), heat-stable toxin (ST) (*est*), colonization factors CS1 through 30, F4, F5, F6, F17, F18, and F41, as well as toxigenic invasion and adherence *loci* (*tia*, *tibAC*, *etpBAC*, *eatA*, *yghJ*, and *tleA*). To construct the database, we revised the existing ETEC nomenclature and used the VirulenceFinder webtool at the CGE website [VirulenceFinder 2.0 (dtu.dk)]. The database was tested on 1,083 preassembled ETEC genomes, two BioProjects (PRJNA421191 with 305 and PRJNA416134 with 134 sequences), and the ETEC reference genome H10407. In total, 455 new virulence gene alleles were added, 50 alleles were replaced or renamed, and two were removed. Overall, our tool has the potential to greatly facilitate ETEC identification and improve the accuracy of WGS analysis. It can also help identify potential new virulence genes in ETEC. The revised nomenclature and expanded gene repertoire provide a better understanding of the genetic diversity of ETEC. Additionally, the user-friendly interface makes it accessible to users with limited bioinformatics experience.

**IMPORTANCE:**

Detecting colonization factors in enterotoxigenic *Escherichia coli* (ETEC) is challenging due to their large number, heterogeneity, and lack of standardized tests. Therefore, it is important to include these ETEC-related genes in a more comprehensive VirulenceFinder database in order to obtain a more complete coverage of the virulence gene repertoire of pathogenic types of *E. coli*. ETEC vaccines are of great importance due to the severity of the infections, primarily in children. A tool such as this could assist in the surveillance of ETEC in order to determine the prevalence of relevant types in different parts of the world, allowing vaccine developers to target the most prevalent types and, thus, a more effective vaccine.

## INTRODUCTION

Enterotoxigenic *Escherichia coli* (ETEC) causes hundreds of millions of cases of infectious diarrhea each year, mainly in developing countries (1), and is ranked number seven on the global burden of 31 food-borne hazards (2). Globally, 1 in 10 child deaths in children under the age of five is a result of diarrheal disease, resulting in 800,000 deaths annually (3). ETEC is also the most common cause of *Escherichia coli* (*E. coli*) diarrhea in farm animals (mainly cattle, pigs, and sheep) (4). The European Food Safety Authority reports that multidrug-resistant *E. coli* is considered an important hazard to public health. The indication associated with most antimicrobial use is post-weaning diarrhea caused by ETEC (5), and recent studies show that there is an increase in antibiotic resistance seen in ETEC infecting humans (6–9). The presence of multidrug-resistant pathogenic bacteria, including ETEC, in raw vegetables and minimally processed fresh vegetables, is a significant public health concern (10).

ETEC bacteria colonize the small intestine via colonization factors (CF), also known as coli surface antigens (CS) or fimbriae (F), and the secretion of enterotoxins, heat-labile toxin (LT) and/or heat-stable toxin (ST), alters the epithelial cell systems (11, 12), resulting in secretory diarrhea and dehydration. Infections can become lethal as a result of severe dehydration and electrolyte imbalance (4) and are a huge problem in many pig herds as well as in humans in geographical regions where access to clean water is limited.

At least 25 antigenically distinct CF types (CFA/I and CS1–CS30 with CS9, CS10, CS11, CS16, CS24, CS25, and CS29 missing, and PCFO71) have been identified and characterized in human ETEC (12–14) and six CFs in ETEC isolated from animals. An additional four adhesins Tia, TibA, EtpA, and EtpB have also been associated with ETEC (12). The enterotoxins LT and ST can be further subgrouped: LTI comprises LTh and LTp [nomenclature is based on human or porcine origin (14)]. At least 28 LTI types have been identified (13; https://github.com/avonm/ETEC_toxin_variants_db). A second enterotoxin, LTII from a buffalo, which activates adenylate cyclase in eucaryotic target cells, was identified in 1983 (15). Further studies found variants of LTII with the proposed designations LTIIa and LTIIb, as well as LTIIc, which was added in 2010 (16). ST in ETEC may be classified into two major subtypes, i.e., STa and STb (17). The plasmid-borne STa gene codes for a 72-amino acid peptide sequence (18). Upon post-translational modification, the highly conserved C-terminal ends (codons 55–72) have been shown to be responsible for the biological activity. Two variants of STa are very similar in structure and function, and the most commonly used nomenclature for these variants are STh (19) and STp (17), based on which host they were originally found in (human and porcine). The mature active STh consists of 19 amino acids and STp of 18 amino acids (20). However, both STh and STp are associated with infections in humans (17) and animals. STa from *E. coli* belong to a family of at least seven types of heat-stable peptide enterotoxins also found in *Vibrio cholerae* non-O1 and O1, *Vibrio mimicus*, *Yersinia enterocolitica*, *Klebsiella pneumoniae,* and *Citrobacter freundii* (18, 21).

The cost of whole-genome sequencing (WGS) has decreased over the last decades, making the technology accessible to routine clinical and microbiological laboratories. Even though WGS provides detailed information that could enable diagnostics and typing based on the information obtained from the bacterial genome, the challenge is to extract the relevant information from the large amount of sequence data that are generated by this technique. Thus, it is important that the data can be interpreted by physicians, veterinarians, and public health professionals, and to achieve this, an ETECFinder database has been constructed and incorporated into the pre-existing web tool, VirulenceFinder, provided by the Center for Genomic Epidemiology (CGE). The web tool is user-friendly and allows for rapid analysis of the obtained WGS data and extraction of relevant information. The ETECFinder contains every known ST, LT-gene, and CFs related to human and animal disease and other genes known to be involved in ETEC virulence/pathogenicity. All accession numbers in the expanded database are available at the CGE website genomicepidemiology / virulencefinder_db / virulence_ecoli.fsa—Bitbucket.

However, ETEC infections pose a major threat to global health in a One Health environment. Rapid diagnostics and accurate classification are of great importance in order to limit the spread of and prevent outbreaks. Hence, there is a need for an updated database to aid the identification and characterization of ETEC isolates. There is no perfect test for ETEC, and it is challenged by the heterogenetic characteristics of the pathogen harboring a broad array of virulence factors, such as the >28 different CFs. Diagnostics of diarrhea caused by ETEC are seldom done in a laboratory but are based on the patient’s or animal’s history and symptoms, thus making monitoring of ETEC very difficult. In surveillance studies, PCR targeting ETEC toxins is mainly used; however, an up-to-date database of virulence factor alleles is important for wider coverage. By expanding the existing VirulenceFinder tool and database, we hope to contribute to a more refined and accurate detection of virulence factors related to ETEC infections in both humans and animals.

## MATERIALS AND METHODS

### Construction of the VirulenceFinder database

The relevant genes and their sequences were compared using BLASTn against the NCBI nucleotide database (https://www.ncbi.nlm.nih.gov/nuccore/) to identify potential matches. Sequences with open reading frames were curated and validated for the presence of only the four nucleotides ATCG. The validated sequences were compared using BioNumerics software (v8.1), and a reference sequence was selected for each allele. The first validated sequence to enter the database was designated as the reference sequence.

To collect alleles, sequences from various papers were compared using BLASTn, and alleles that matched 80% sequence identity and total length were included in the database. Partial genes were excluded from the database to ensure completeness of sequences. The database is stored as a text file in FASTA format with a unique identifier for each reference sequence followed by its sequence, which can be downloaded from the CGE website (genomicepidemiology / virulencefinder_db — Bitbucket).

### Creation of the ETECFinder database and update of the VirulenceFinder database

The original VirulenceFinder database already contained 14 genes (original designations: *cfa_c, cofA, f17-A, f17-G, fanA, fasA, fedA, fedF, K88ab, fimF41, lngA, ltcA*, *sta1,* and *stb*) (see Table S1) associated with ETEC. To expand the database, the full *loci* for CFs CFA/I, CS8, and CS21, as well as animal-specific fimbriae F4, F5, F6, F17, F18, and Fim41, were added. The genes and *loci* were revised, updated, and added (see Table S1). The original designation *ltcA*, where “c” stands for chicken (22), was deleted.

**Fig 1:**
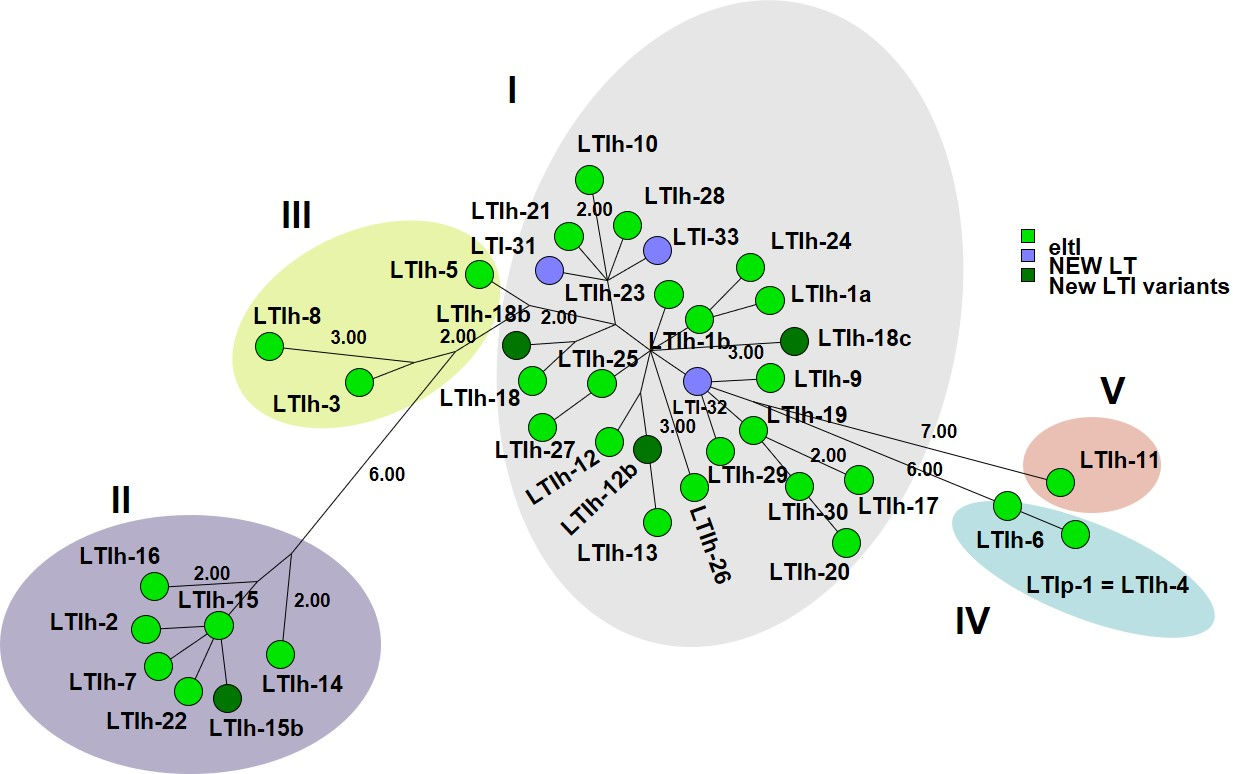
Maximum parsimony tree of 31 LTI reference nucleotide sequences, three new LTI types (LTI-31, LTI-32, and LTI-33), and four new LT variants (LTIh-12b, LTIh-15b, LTIh-18b, and LTIh-18c) identified in 890 ETEC genomes analyzed with the revised VirulenceFinder database. The colors and groups I–V correspond to the designation and colors in Joffré et al. (13), where concatenated protein sequences were presented. Four nucleotides “TGAA” were removed from the NCBI-submitted sequences as described in the text. Only branch lengths larger than 1.00 are shown.

The LT toxins presented a challenge when using nucleotide sequences. The original definitions of LT types were based on the specific combination of the A and B subunits (Table S2). For instance, LTIh-3 and LTIh-5 have identical A subunits, as do LTIp-1, LTIh-4, and LTIh-6. The same applies for the B subunit of LTIh-9, LTIh-11, and LTIh19; LTIh-17, LTIh-20, LTIh-29, and LTIh-30; LTIh-3 and LTIh-8; LTIh-2, LTIh-7, LTIh-15, LTIh-16, and LTIh-22; LTIh-1a, LTIh-1b, LTIh-10, LTIh-12, LTIh-13, LTIh-18, LTIh-21, LTIh-23, LTIh-24, LTIh-25, LTIh-26, LTIh-27, and LTIh-28, respectively. However, the reference sequences submitted to NCBI vary in whether or not the nucleotide sequences encoding the holotoxin include the overlap of the stop codon for the sequence encoding the A subunit and the start codon encoding the B subunit. Sequences *eltI-*2, *eltI-*17, *eltI-*18, *eltI-*19, *eltI-*20, *eltI-*21, *eltI-*22, *eltI-*23, *eltI-*24, *eltI-*25, *eltI-*26, *eltI-*27, and *eltI-*28 have no overlap and are 1,152 bp, whereas the remaining *eltI* sequences (*eltI-*1a, *eltI-*1b, *eltI-*3, *eltI-*5, *eltI-*6, *eltI-*7, *eltI-*8, *eltI-*9, *eltI-*10, *eltI-*11, *eltI-*12,1 *eltI-*3, *eltI-*14, *eltI-*15, *eltI-*16, *eltI-*29, and *eltI-*30) are 1,148 bp. Due to the high sequence identity between the *eltI (elth*) sequences (>98.5%), the four base pair difference may result in a wrong *eltI* type for tested sequences using the CGE VirulenceFinder tool. As an illustration, *eltI-*1 (1,148 bp with an overlap) has a non-synonymous SNP at position 88 compared to *eltI-*25 (1,152 bp with no overlap). To address this difference, we removed the “TGAA” nucleotides from the *eltI* reference sequences (*eltI-*2, *eltI-*17, *eltI-*18, *eltI-*19, *eltI-*20, *eltI-*21, *eltI-*22, *eltI-*23, *eltI-*24, *eltI-*25, *eltI-*26, *eltI-*27, and *eltI-*28) in our database. The final revision now includes the *eltI*AB sequence that encodes the holotoxin for 30 human LTIh types (LTIh-1 through LTIh-30), with two LTIh-1 alleles and one porcine LTIp type (LTIp-1). Agreement between the phylogeny using maximum parsimony analysis on these nucleotide sequences with the designations and colors of concatenated protein sequences by Joffré et al. (13) was then tested (see Fig. 1). Additionally, we identified and analyzed 15 holotoxin sequences for LTII (*eltII*AB) and added them to the database. These sequences consist of one LTII-a, two LTII-b, nine LTII-c, two LTII-d, and one LTII-e.

To account for the variations in the porcine variant of STa1, we replaced the two *sta1* alleles with 15 *estap* alleles. For the human variant of STah2 and STah3, we added four *estah* alleles. Furthermore, we renamed three *stb* alleles for the porcine variant STb1 as *estb* and added one variant of ST2b to the database (see Table S1).

We used a beta version of the VirulenceFinder database, which includes the ETECFinder database (ETEC-related genes), to search for virulence genes in 1,083 preassembled *E. coli* genomes. The ETECFinder database is designed to perform a genotypic detection of ETEC virulence genes, and all genes and alleles in the database are identified with a GenBank accession number or identifier (ID). To simplify the LT typing, we concatenated the *eltA* and *eltB* sequences and removed the overlapping four bases present in a subset of the LT types. We then analyzed the nucleotide sequences that encode the LT holotoxin. Additionally, we ran the 1,083 genomes through the CGE web tool SerotypeFinder, CGE Server (dtu.dk).

Genomes were defined as ETEC if they were positive for any combination of the genes *estah*, *estap*, *estb*, *eltI*AB, or *eltII*AB. Additionally, genomes were classified as ExPEC_JJ_ if they were positive for two or more of *papAH* and/or *papC* (P fimbriae), *sfa/focDE* (S and F1C fimbriae), *afa/draBC* (Dr-binding adhesins), *iutA* (aerobactin siderophore system), and *kpsM* II (group 2 capsules) (23), and as UPEC_HM_ if they were positive for two or more of *chuA* (heme uptake), *fyuA* (yersiniabactin siderophore system), *vat* (vacuolating toxin), and *yfcV* (adhesin) (24).

### Data set and data used for the validation of the ETECFinder database

A collection of 1,083 ETEC isolates, representing the global diversity of ETEC, was selected for sequencing as part of various studies conducted between 1980 and 2011. These isolates were sourced from 57 different countries across Asia, Africa, and North, Central, and South America. Among the 1,083 ETEC genomes sequenced, 362 genomes had been previously described, while the remaining genomes were included to expand the existing collection and fill knowledge gaps regarding CF profiles. The selection of isolates was based on their host and virulence profiles, ensuring representation across the spectrum of ETEC diversity. These encompassed isolates obtained from adults (both indigenous populations and travelers), children under 5 years of age, and farm animals infected with ETEC. Although the data set may be biased toward ETEC collected from humans and pigs, the ETECFinder database will be continuously updated with contributions from the ETEC research community. As more research is conducted and new isolates are collected from different hosts, the database is expected to expand and include a broader range of host-associated ETEC virulence factors. The ongoing input from the research environment will ensure that the ETECFinder database remains comprehensive and reflective of the evolving understanding of ETEC diversity across various hosts. Additionally, the data set covers the 20 known ETEC lineages associated with human disease previously described in von Mentzer et al. (25) and, together with additional genomes, it aims to cover ETEC diversity on a global scale as well as multiple hosts (humans and animals). (Fig. S1–S3; Data Set S1). A subset of the clinical samples was collected from asymptomatic individuals. The isolates were screened for toxins using PCR and a subset of CFs using dot blot with available antibodies. A subset (*n* = 362) of the genomes was manually analyzed for virulence profiles using comparative genomics (25). The additional 721 genomes followed the same protocols for DNA extraction, sequencing, and assembly as described in von Mentzer et al. (25). An additional level of quality control of the reads and assemblies was performed to ensure high-quality sequencing data. FASTQC v0.11.8 (Babraham Bioinformatics - FastQC A Quality Control tool for High Throughput Sequence Data) and MultiQC (26) were used to investigate read quality and GC content (between 49% and 51%). Kraken/bracken was used to identify potential contamination in combination with assembly statistics, such as species abundance (>65% *E*. *coli*), the total number of bases (4.5–6 MB), and the total number of contigs (<300), and the *N*_50_ value (>30 kb) was used to further assess the quality of the assemblies. The two BioProjects (PRJNA421191 with 305 and PRJNA416134 with 134 sequences) plus the reference genome of ETEC TW11681 (accession no. AELD00000000) analyzed by Hazen et al. (27) were also tested using the revised VirulenceFinder database. The genome of the widely used ETEC reference strain H10407 (chromosome, accession no. FN649414, and plasmids FN649415.1_p52, FN649415.1_p58, FN649415.1_p666, and FN649415.1_p948) was also tested.

## RESULTS

Including original, renamed, replaced, and new genes, the beta ETECFinder database contains 524 alleles representing 38 *loci*; the gene name, the number of new alleles, and the CFs or protein names are listed in Table S3. These alleles were added to and/or changed in the existing database at the CGE website.

### Updated nomenclature for enterotoxins

When constructing the ETECFinder database, a search for the various ST and LT types was conducted, which revealed identical genes but with different nomenclature. To ensure that identical gene sequences do not have multiple names, a revised, new nomenclature is proposed and listed in Table 1, along with the present nomenclature and accession numbers and/or references of the listed proteins and genes.

**TABLE 1:** Nomenclature of LT and ST toxins and genes

Previous synonym for toxin type (accession no.; reference)	New synonym for toxin [adapted from reference (28) and this study]	Previous synonym for toxin gene (accession no.; reference)	New synonym for toxin gene (this study)	Length of amino acid sequence(s)
STa				
ST_A_ (29) STIa (STp) [WP_001353651.1 (27)] ST-IA/ST-P (BAI49232) st-ia/st-p (BAT57149) STa1-STa6 (28) STI (18) STp (17)	STap1, STap4, STap5, STap6, STap7, STap8, and STap9	*estA* (29) *sta1* = *estIa* *estIa* [located on plasmid AP010910 (30)] *est* [located on plasmid AP014654.2 (31)] *estA1* (32) *estA4* [J03311.2 (33)] *estP* (20)	*estap* ^*a*^	Precursor: 72 Biological activity: 18
STIb (STh) [WP_023485648.1 (27)] STa (18) STa1-STa6 (28) STh (19)	STah2 and STah3	*sta2* [WP_023485648.1 (27)] *st* [M29255 (34)] *estA2* [M18345.1 (33)] *estA3* [M18346 (33)] *estH* (20)	*estah* ^*a*^	Precursor: 72 Biological activity: 19
STb				
ST_B_ (29, 35) STb [AAK29747.1 (36)] STI (18, 36)	STb1	*estB* (35) *Stb* (AY028790)	*estb* ^*b*^	Precursor: 71 Biological activity: 48
STII [OTB52774.1 (37)]	STb2	Found in NEMH01000033.1:c26982-26767	*estb* ^*b*^	
LTI				
LT LT1–LT28 (25) lt-a, porcine, ltp-a [CBJ04426.1 (38)] LT1 (hLT) and LT4 (pLT) (39) LT-I (40, 41) LTh A and LTh B (42) LTh-I (40, 43)	LTIh-1–LTIh-30 LTI-31–LTI-33	*toxA* and *toxB* (44) *toxL1* and *toxL2* (45) *eltI* *eltA, eltB* [FN649417 (14)] *eltI*h *eltI*ABh	*eltIAB*-1– *eltIAB*-33^*c*^	A subunit: 258 B subunit: 124
LTIp (42), LTp-I (40, 43) LT4 = LTA [ABV01312.1 (14)] and LTB [ABV01313.1 (14)]	LTIp-1 = LTIh-4	*eltI*p *eltA, eltB eltI*ABp *eltA,* and *eltB* (EU113243.1)	*eltIAB*p-1	A subunit: 258 B subunit: 124
LTII^*d*^				
LT-II (41) LT-IIa A (AAA24093.1) LT-IIa B (AAA24094.1) LT-IIa (40, 43, 46)	LTII-a	[JQ031711 (47)]	*eltIIAB*-a	A subunit: 259 B subunit: 124
LT-IIb (40, 43, 46) LT-IIb A (AAA53285.2) LT-IIb B (AAA53286.1)	LTII-b1 LTII-b2	[JQ031712 (47)]	*eltIIAB-*b Two variants	A subunit: 263 B subunit: 122
LTIIc (16, 47) LT-IIcA and LT-IIcB (16) LT-IIc1–LT-IIc6 [JQ031705–JQ031710 (46)]	LTII-c1–LTII-c9	[JQ031705 (47)]	*eltIIAB-*c Nine variants	A subunit: 259 B subunit: 122
LTIId (AP019856) LT-IId A (BBM81071.1) LT-IId B (BBM81072.1)	LTII-d1 LTII-d2	*eltA* (found in AP019856.1:4740935–4741696) *eltB* (found in AP019856.1:4741709–4742080)	*eltIIAB-*d Two variants	A subunit: 253 B subunit: 123
LT IIA (HAL0808134.1) LT II B (HAL0808133.1)	LTII-e	(found in DABMTW010000030.1:20190–20561)^*e*^ (found in DABMTW010000030.1:c20561-20190)^*e*^	*eltIIAB-*e	A subunit: 263 B subunit: 123

^*a*^ Followed by the specific toxin subtype designation, i.e*.*, *estap*-STap1, *estah*-STah2, etc.

^*b*^ Followed by the specific toxin subtype designation, i.e., *estb*-STb1 and *estb*-STb2.

^*c*^ Three new *eltIAB* subtypes and four new *eltIAB* variants were identified in this study.

^*d*^ *eltIIAB* sequences were 48.6%–54.3% identical to *eltIAB* sequences (Fig. 2).

^*e*^ REGION: 20190-21333.

**Fig 2:**
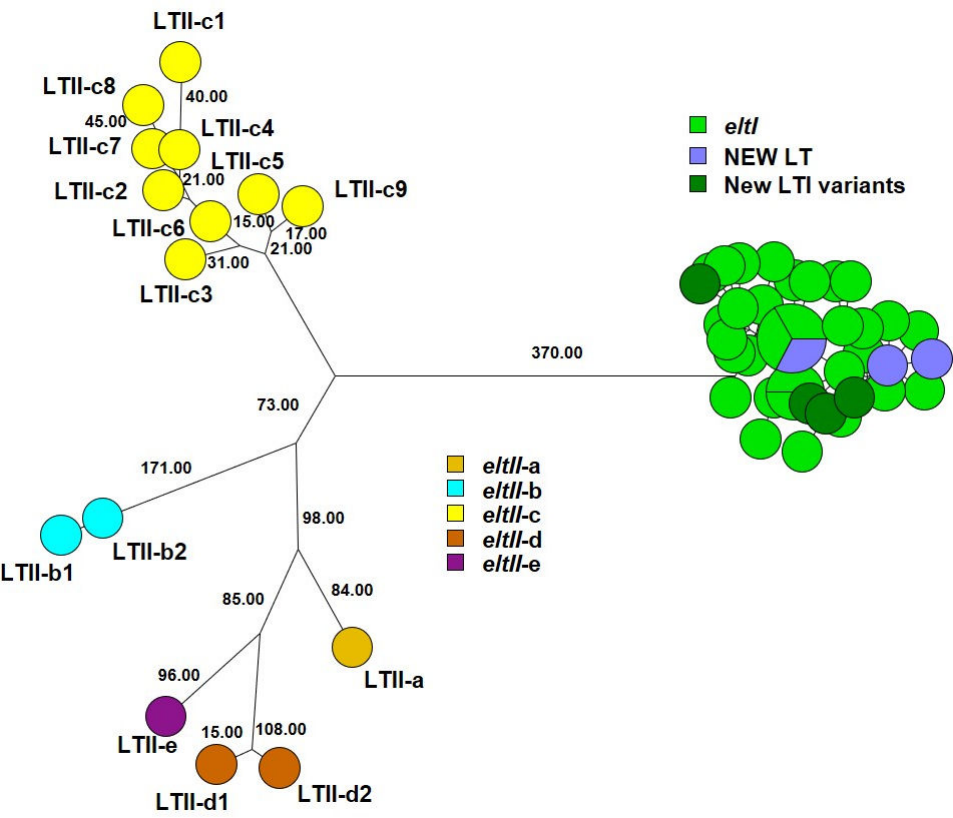
Maximum parsimony tree of 31 LTI and 15 LTII reference nucleotide sequences, three new LTI types (LTI-31, LTI-32, and LTI-33), and four new LTI variants (LTIh-12b, LTIh-15b, LTIh-18b, and LTIh-18c) identified in 890 ETEC genomes analyzed with the revised VirulenceFinder database. LTII-a–LTII-e sequences were 48.6%–54.3% identical to LTI sequences. Only branch lengths of 15.00 or larger are shown. For a high resolution of *eltI*, see Fig. 1.

As a result of the new nomenclature, the existing VirulenceFinder web tool had to be revised. The revision led to changes in the nomenclature of *est* and *elt* genes, encoding ST and LT, respectively, and the number of alleles. Some ETEC virulence genes were previously included in the VirulenceFinder database. However, the database did not contain genes from the entire fimbriae but merely one or two genes. As a result of this study, genes from the entire CF *loci* have been added to the database. Changes in the VirulenceFinder database are listed in Table S1 and new alleles in Table S3.

### Implementation of ETECFinder

**Fig 3:**
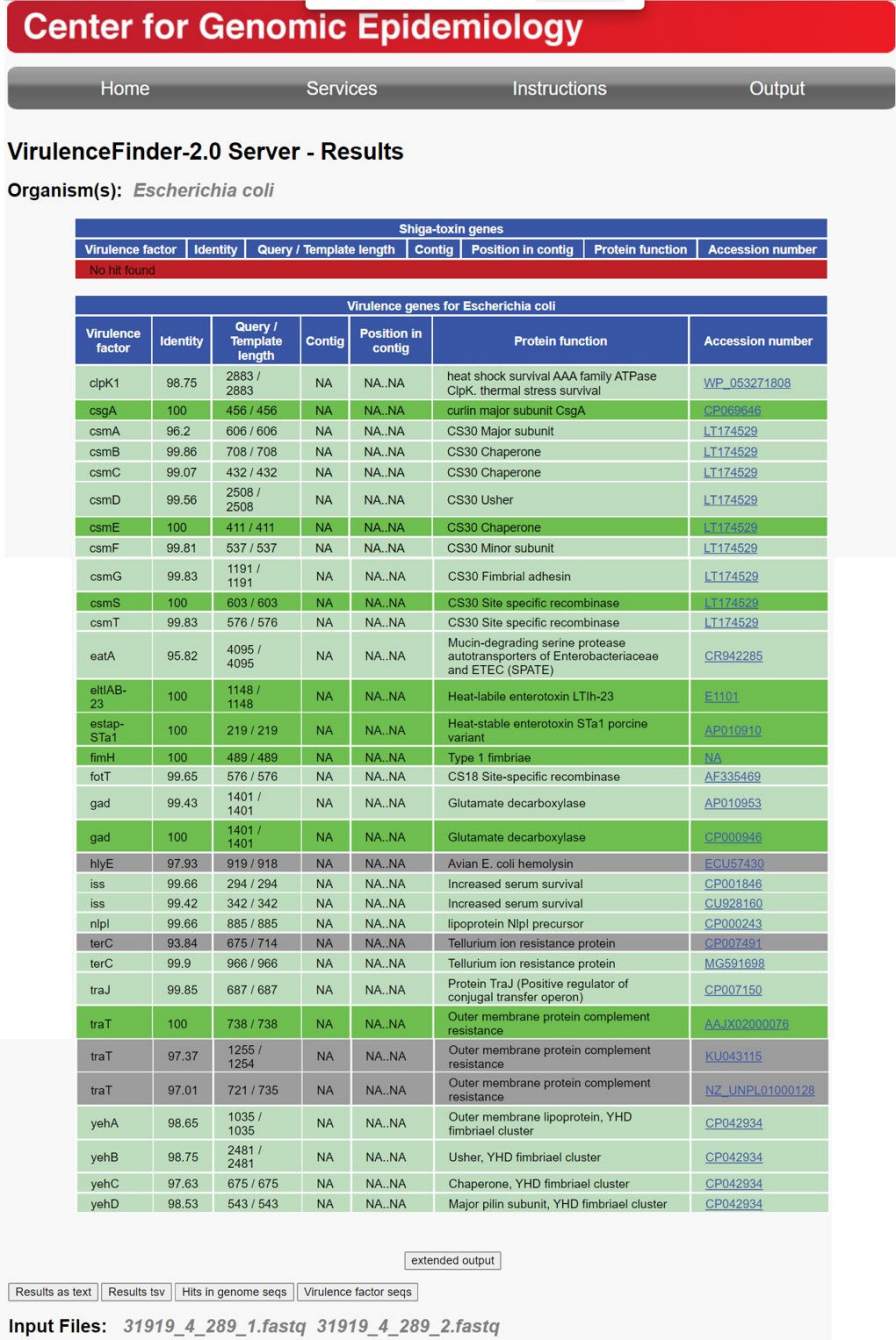
ETECFinder results for an enterotoxigenic *Escherichia coli* (strain M2, ID 31919_4_289) isolate in the short output format using the revised VirulenceFinder database with FASTQ files from the same enterotoxigenic *Escherichia coli* as in Fig. 4. Multiple new virulence gene alleles are identified. Shown are the names of the best-matching allele in the VirulenceFinder, the percentage of nucleotides that are identical to the best-matching allele in the database and the corresponding sequence in the genome (percent identity), the length of the alignment between the best-matching allele in the database and the corresponding sequence in the genome [also called the high-scoring segment pair (HSP)], the length of the best-matching allele in the database, the name and function of the best-matching allele, and an LT type. Color indications: the dark green color indicates a perfect match for a given gene. The percent identity is 100%, and the sequence in the genome covers the entire length of the virulence gene in the database. The light green color indicates a warning due to a non-perfect match, percent identity < 100%, HSP length = virulence gene length. The gray color indicates a warning due to a non-perfect match, HSP length is shorter than the virulence gene length, percent identity = 100%. The red color indicates that no virulence gene with a match over the given threshold was found. See VirulenceFinder 2.0 output (dtu.dk).

**Fig 4:**
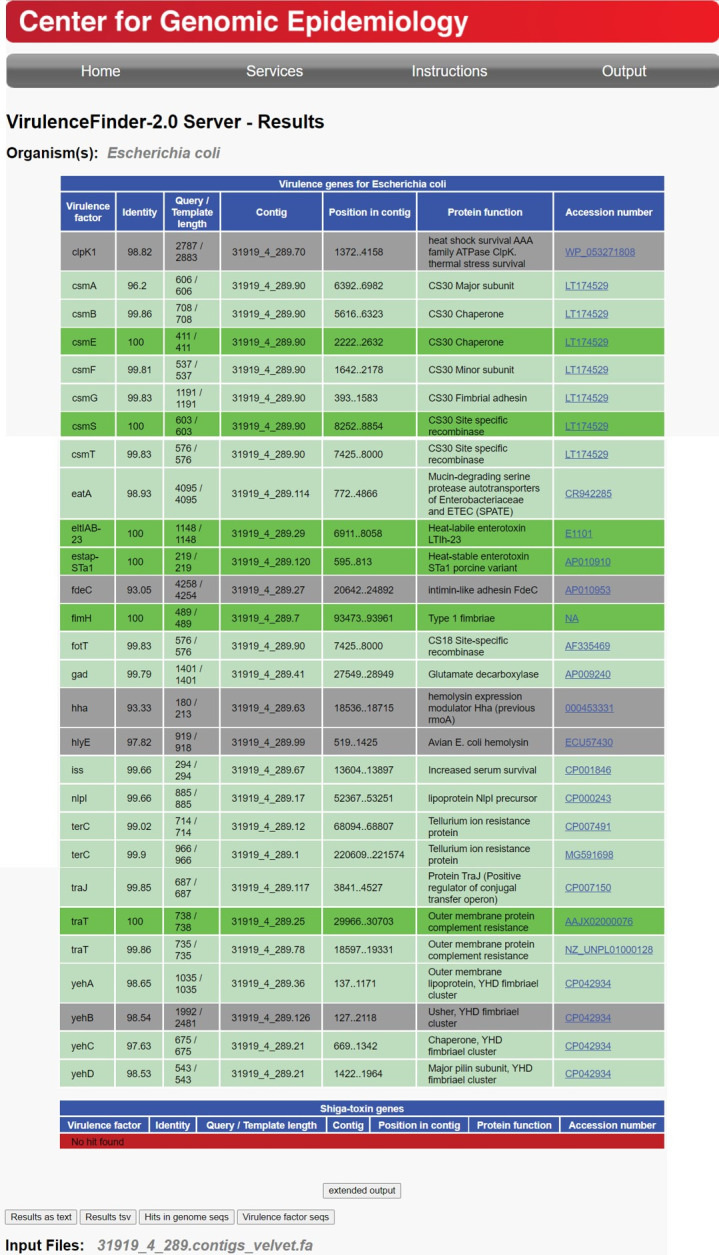
ETECFinder results for an enterotoxigenic *Escherichia coli* isolate in the short output format using the revised VirulenceFinder database with a preassembled genome of the same enterotoxigenic *Escherichia coli* genome as in Fig. 3.

The ETECFinder database comprising all known ETEC virulence genes is now a part of the VirulenceFinder tool, which can either take reads from Illumina, Ion Torrent, Roche 454, SOLiD, Oxford Nanopore, and PacBio or assembled sequences as input. Because the CGE web tools only allow for one sequence at a time, a script for batch analyses was kindly provided by bioinformatician Maja Weiss (CGE) so that batch analyses could be performed on the SSI server. First, raw data generated from sequenced and assembled bacterial genomes were used as input. By performing a BLAST search of the genome against the database, the closest matching alleles were identified, and a virulence profile was determined. The CF profile is based on the various alleles found. The short output format includes the identified best-matching ETEC allele in the database. An additional extended output includes the nucleotide sequence of the ETEC alleles identified. For a few selected outputs, both types of input data—reads and assembled genomes—were analyzed and compared. Here, we discovered different outputs/results from using assembled genomes compared to read data. To showcase this, the genome of the ETEC strain positive for CS30 as well as LT and STp was analyzed using the ETECFinder/VirulenceFinder database using both types of data. VirulenceFinder identified the whole CS30 gene cluster (*csmS, T, A, B, C, D, E, F,* and *G*) and both toxin genes but did not identify genes *fdeC* and *hha* (Fig. 3) when the reads were used as input. Conversely, when analyzing the assembled genome, VirulenceFinder failed to identify the full CS30 gene cluster, missing *csmC* and *csmD*. However, both *fdeC* and *hha* were identified (Fig. 4). We therefore contacted the CGE helpdesk in order to inquire about these differences in output. Hence, it was discovered that the old version of VirulenceFinder did not allow for nucleotide overlap in the assembled contigs. The genes *csmC* and *csmB* are slightly overlapping with 20 nucleotides, and the genes *csmD* and *csmE* overlap with four nucleotides. This was amended, and the results presented in this paper reflect the results of the revised VirulenceFinder, which now allows overlaps of up to 30 nucleotides, as is also the default for ResFinder at the CGE website. An option was added to the command line tool, allowing the users to tweak this value. Thus, an additional 1,051 genes—primarily colonization factor—were detected, as were *tibA* and *tibC*, which overlap by eight nucleotides (Data Set S1 row CB1114:UL1114, summarized in Table S9).

When the KMA read aligner sees too many mismatches within a certain area, it will not align anything to that area (R. S. Kaas, DTU, personal communication), and the end of *fdeC* has a lot of mismatches, whereas the beginning of the gene aligns perfectly. The contig alignment indicating 93.05% identity in the assembled contig is a bit misleading as all the mismatches are found toward the end of the gene. The actual identity is significantly lower at 71.89%. Likewise, for the *hha* gene, the identity is 76.73%. The cutoff value on the KMA web tool using reads is at least 85%, which is the reason why there are no hits for *fdeC* and *hha* when using reads as input.

### Validation of the ETECFinder database

The revised database was tested on a collection of 1,083 preassembled genomes (sequenced by Astrid von Mentzer) to predict the virulence profile and compared to previous bioinformatics analyses. The threshold for a positive hit was set at 90% sequence identity and minimum length at 60%. This resulted in 890 ETEC matches (i.e., enterotoxin-positive *E. coli*) and 193 non-ETEC matches (i.e., enterotoxin-negative *E. coli*). In addition to identifying ETEC virulence genes, the VirulenceFinder searches for genes used for *E. coli* pathotype diagnostics. Interestingly, 18 genomes were predicted as hybrid pathotypes, 13 ExPEC_JJ_-UPEC_HM_, 4 ETEC/ExPEC_JJ_, and 1 ETEC-UPEC_HM_. A total of 2 genomes were predicted as UPEC_HM_, 15 as ExPEC_JJ_, and 7 as enteroaggregative *E. coli* (EAEC), two of which were EAEC-ExPEC_JJ_. One hundred fifty-six were negative for genes used for defining any *E. coli* pathotypes (EAEC, EIEC; EPEC, ETEC, ExPEC_JJ_, and UPEC_HM_).

### Enterotoxins

To further investigate the different ETEC toxins identified in the data set, we report that the most prevalent toxin profiles (here using the newly proposed nomenclature) were STah-only (193/1,083; 18%), LT-only (184/1,083; 17%), LT + STah (157/1,083; 14%), STap-only (100/1,083; 9%). Additional toxin profiles with LT in combination with other toxins were LT + STah (157/1,083; 14%), LT + STap (109/1,083; 10%), LT + STb (107/1,083; 10%), LT + STap + STb (18/1,083; 1.7%), and LT + STah + STap (8/1,083; 0.7%). A new variant of STb was discovered in one genome. Thirteen genomes had a combination of STap1 and STb1. STap1 and STah3 were only found in combination with LT18 in eight genomes of serotype O148:H28. The most common combination of LT and ST subtypes was LTIp and STb1 (107/1,083; 10%), followed by LT30 and STah3 (90/1,083; 8%) (Table S4). Three new LT types were discovered, where LT32 was identified in 94 genomes (51 alone and 15 together with STap1), followed by LT33 (12 alone and 15 with STap1) and LT31 with STap1 (1). Additional new variants designated LT15b (11/1,083), LT12b (2/1,083), LT18b with STap1 (6/1,083), and LT18c with STap1 (1/1,083) were found in the analyzed genomes (Fig. 1).

Not all genes were found with 100% identity, indicating alterations in the sequence compared to the reference sequence found in the database. This could be a result of point mutations or poor sequencing.

### Human-specific ETEC colonization factors

Five hundred twenty-nine (48%) of the genomes used in this study were positive for at least one CF gene in two to five different CF types (regulator, major subunit, minor subunit, chaperone, usher, and adhesin). The specific allele combination findings are described in detail in Table S5. One hundred ninety-seven genomes were positive for only one CF gene (specific single allele findings are described in detail in Table S6). CFA/I encoded by *cfaA*, *cfaB*, *cfaC*, *cfaD,* and *cfaE,* of which 80 genomes contained the full CFA/I locus, was found in combination with other CFs in 327 genomes (Table S5). In three ETEC-UPEC_HM_ genomes of serotypes O128ac:H45 (two genomes) and O153:H46 (one genome), CFA/I was the only CF (Table S6). The specific allele combination findings are listed in Table S5 and Data Set S1. In summary, 529 genomes contained 33 different combinations of colonization factors, with CFA/I being the most commonly found in 338/529 (63%) genomes, and 182/338 (54%) of these were also CS21 positive. Furthermore, CS21 was also found with other combination CFs such as CS6, 39/529 (7%) and alone in 32/197 (16%), followed by CS12 in combinations in 64/529 (12%) and alone in 57/197 (29%).

### Animal-specific ETEC alone or in combination with human colonization factors

None of the genomes contained the full clusters for F4, F6, or F18. Several putative gene clusters were identified with genes from different colonization factor gene clusters in a total of 138 genomes. F4 genes were found in 129 sequences, *faeA* (74), *faeB* (93), *faeC* (109), *faeD* (32), *faeE* (127), *faeF* (117 once and 2 sequences twice), *faeGab* (6), *faeGab1* (6), *faeGab2* (0), *faeGac* (79 once and 5 times twice), *faeGad* (2), *faeG* (0), *faeH* (107 once and 1 twice), *faeH1* (0), *faeH2* (0), *faeI* (111), *faeI1* (1), *faeI2* (0), and *faeJ* (95 once and 5 twice). ), *faeD* (32), *faeE* (127), *faeF* (117 once and 2 sequences twice), *faeGab* (6), *faeGab1* (6), *faeGab2* (0), *faeGac* (79 once and 5 times twice), *faeGad* (2), *faeG* (0), *faeH* (107 once and one1 twice), *faeH1* (0), *faeH2* (0), *faeI* (111), *faeI1* (1), *faeI2* (0), and *faeJ* (95 once and 5 twice). One-hundred- and-sixteen Of these 129, 116 were in combination with F5 and *fimF41* (11), F4-F6-F17 (12), F4-F17 (84) and 2,020 were in combinations with human colonization factors. F4-CS23 genes were found in six genomes. Additional combinations of F4 and CS23 genes with human and other animal colonization factor genes were found in 14 genomes: specifically, F4-F5- *fimF41*-CS23 (6), F4-CS12-CS23 (3), F4-CS12-CS23-CS26 (3), F4-CS12-CS20-CS23 (1), and F4-F17-CS23 (1). Out of these 20 F4-(CSxx)-CS23 combinations, 15 were predicted as ETEC, one as ExPEC_JJ_, one as UPEC_HM_, one as ExPEC_JJ_/UPEC_HM_, and two could not be assigned to a pathotype. Other combinations included F4-F17 (84), F4-F6-F17 (12), F6-CS8 (2), F6-CS8-F17 (1), F6-F18-F17 (1), and F18-F17 (18) (Table S7). The genes were most often located on the same contig indicating that this gene cluster on that specific contig may encode a putative colonization factor. Of the 129 F4 genes, 14 were found in non-ETEC sequences (Table S7). F5 (*fanABCDEFGH*) was complete in six sequences in combination with F4, *faeCDEFHI* (6), *fimF41* (6), and CS23 *aalH* (6) genes. F6 was found in 18 sequences, *fasAB* (17), *fasC* (0), *fasDEFG* (16), and *fasH* (15). Thirteen of these were in combination with other F genes, F4 (2), F4ac (10), and F18ac (1). F18 genes were found in 33 sequences, *fedAab* (7), *fedAac* (34), *fedAnt* (0), *fedBEF* (33), and *fedC* (7). Twelve of these were found in ETEC-ExPEC_JJ_/UPEC_HM_-negative genomes and one in combination with F6-F17.

### Additional ETEC non-canonical virulence genes

The genes for enterotoxigenic *E. coli* autotransporter A (*eatA*, three alleles), extracellular serine protease EspC (also called EPEC enterotoxin) (*espC*, three alleles), and the plasmid-encoded type II secretion pathway-related protein EtpD (*etpD* three alleles) were already included in the original VirulenceFinder database (48). The gene *eatA* was found once in 392 and twice in 2 genomes, and *espAC* was found once in an *eae*-positive sequence of *in silico* serotype O71:H49. The genes *etpA*, *etpB,* and *etpC* were found, respectively, in 31, 340, and 341 sequences. Of note, none of these were also positive for *etpD* found in eight sequences. The adhesin gene *tia* was found once in 182 genomes and twice in 1 genome. Additionally, genes such as *tibA* and *tibB* were found in 113 and 131 sequences, *tleA* was found once, and *yghJ* was found once in 869 and twice in 79 genomes.

### Validation of the ETECFinder database on the ETEC collection in BioProjects PRJNA421191 and PRJNA416134

The revised database was used on a total of 440 genomes from BioProjects (PRJNA421191 with 305 and PRJNA416134 with 134 genomes) plus the reference genome of ETEC TW11681 (accession no. AELD00000000) listed by Hazen et al. (27). The toxin profile for the 269 genomes listed in Hazen et al.’s (4) Table S1 was initially determined using BLASTN large-scale BLAST score ratio (LS-BSR) analysis and represented a total of 346 accession numbers (S1, RAW data on the 269 genomes). The results on each of these accession numbers were “Moved to match” each of the genomes in Hazen et al.’s Table S1 (4). We then compared the published toxin and CF profiles from Hazen et al. with the output from analyzing the same genomes with the VirulenceFinder. The toxin profile covering *eltIAB* and *est* matched between the LS-BSR and VirulenceFinder approach for all of the 269 analyzed genomes. Furthermore, using the VirulenceFinder allowed us to determine the toxin subtype for each of the genomes: 145 genomes were *estah*-STah2, 82 *estah*-STah3, one each of *estap*-STap1, *eltIAB*-30; *estah*-STah2, *eltIAB*-15; *estah*-STah3, *eltIAB*-22; *estah*-STah3, *eltIAB*-29; and *estah*-STah2. Combinations of two toxins *estah-*STah2 and *estah-*STah3 were found in 11, *estap-*STap1 and *estah-*STah2 in 1, and *estap-*STap1 and *estah-*STah3 in 1 genome. Twenty-four genomes were toxin negative.

CF profiles determined by VirulenceFinder matched with LS-BSR in 237 genomes: CFA/I (defined as positive for four out of five *cfa* genes) + CS21 (defined as positive for 14 out of 16 *lng* genes) was found in 125 genomes, CS5 (five out of six *csf* genes; *csfE* was not detected in any of the genomes) + CS6 (three out of four *css* genes) was found in 56 genomes, CS6 + CS21 was found in 24 genomes, CF-negative (20), CS21 (5), CFA/I (4), CS6 (2), and CS22 (1) were also found by VirulenceFinder. In 130 genomes, two copies of *cfaD* (CFA/I) were found. In PNTM01000001.1, the full CFA/I encoding cluster (*cfaABCDE*) was found split across three different contigs (PNTM01000137.1 , PNTM01000154.1, and PNTM01000058.1), see Data Set S1 and Table S8.

Additional CF genes that were not identified by LS-BSR or mentioned in Hazen et al. (4) were found in 26 genomes and included single genes *cfaD* (7), *csaD* (13), *csfA* (1), *lngHIJP* (2), *lngP* (1), and *cswR* (2). In six genomes, *cfaD* was found by VirulenceFinder instead of *lng genes* for CS21 found by LS-BSR. These findings are shown in Data Set S1 and summarized in Table S8.

Two sequences that had been found negative for ST and CFs by LS-BSR fulfilled the criteria for both ExPEC and UPEC (PNRS00000000 and PNVD00000000). Four sequences (PNRS00000000, PNVC00000000, PNVD00000000, and PNYQ00000000) were enteroaggregative *E. coli* (EAEC) being positive for *aggR* and one of four different AAFs (AAFI, AAFIII, AAFIV, and AAFV). Additional results for the analyzed 269 sequences as well as the remaining 93 sequences not analyzed by LS-BSR can be found in the tables in Data Set S1.

### Validation of the ETECFinder database on the ETEC reference strain H10407

Using the revised database on the annotated sequences for ETEC reference strain H10407 (chromosome, accession no. FN649414, and plasmids FN649415.1_p52, FN649415.1_p58, FN649415.1_p666, and FN649415.1_p948/NC_017724.1) identified the following virulence genes: *astA, csgA, fdeC*, *fimH, fyuA, gad, hha*, *hlyE, irp2, nlpI, shiA*, *terC, terC, tia*, *tibA, yehA, yehB, yehC, yehD,* and *yghJ*, on the chromosome. Additional virulence genes *anr, astA,* the full CFA/I locus [*cfaA, cfaB, cfaC, cfaD* (two copies)*,* and *cfaE*]*,* gene encoding STah2 (*estah*-STah2) along with the non-canonical virulence genes *eatA* and the genes encoding the Etp two-partner secretion system and associated glycosyltransferase (*etpBAC*) were identified on plasmid p948. The two copies of *cfaD* were both found on plasmid p948 at 100% identity to accession numbers FN649418 (length 375 bp) and M55661 (length 435 bp) at positions 89,363–89,737, and 43,366–43,800, respectively. On plasmid p666, genes encoding LTh (*eltIAB*-1) and STap1 (*estap*-STap1) were identified. Plasmids p52 and p58 did not harbor any virulence genes. Thus, all previously described virulence genes in H10407 were identified with the ETECFinder database.

## DISCUSSION

The decreasing costs of whole-genome sequencing have led to an increase in bacterial pathogen sequencing. However, extracting the correct data for analysis from WGS data can be challenging, despite its increased availability to routine diagnostic laboratories. To address this issue, we developed, implemented, and evaluated an ETECFinder database to predict ETEC-associated virulence genes based on our own WGS data and on two published BioProjects PRJNA421191 with 305 and PRJNA416134 with 134 sequences. This database has been integrated into the pre-existing VirulenceFinder, which is accessible at www.cge.cbs.dtu.dk/services/VirulenceFinder/. Users can upload preassembled bacterial genomes or short sequence reads, and the CGE web tools have been designed to facilitate use and output for users with limited bioinformatics experience.

Using the revised VirulenceFinder database to analyze 1,083 preassembled genomes, we identified 890 ETEC sequences, while 193 sequences were negative for enterotoxin genes. All sequences had originally been identified as ETEC-positive by PCR, and the failure to detect ETEC genes in 193 genomes could be explained by loss of toxin genes (which are often plasmid-encoded and therefore can be lost during storage) or by poor sequencing and assembly quality. A similar failure to reproduce PCR results with WGS was also observed by Hazen et al., where 26 PCR-positive results could neither be confirmed by LS-BSR nor by VirulenceFinder. However, VirulenceFinder did identify CFA/I in four genomes that were CFA/I-negative by LS-BSR and four *cfaD*-positive genomes that were CS6-positive by PCR but CS6-negative by LS-BSR. Additionally, VirulenceFinder found genes for five different CFs that had not been found by LS-BSR. The enterotoxin profile covering *eltIAB* and *est* matched between the LS-BSR and VirulenceFinder for all of the 269 analyzed genomes. Furthermore, using the VirulenceFinder allowed us to determine the toxin subtype for each of the genomes and identify 13 genomes with more than one copy of an enterotoxin gene.

Detecting colonization factors in ETEC is challenging due to their large number, heterogeneity, and lack of standardized tests. However, the detection of LT and ST defines an ETEC isolate, although many such isolates may express colonization factors specific to either animals or humans. In this study, we found 138 sequences with different combinations of human and animal colonization factor genes primarily involving F4 and F18. Further investigation of eight selected genomes harboring a combination of different CF genes revealed that the minor fimbrial subunit encoded by *aalH* (CS23) was located on the same contigs as F4 genes *faeH* and *faeI*. As *aalH* has been described as similar to *faeJ* (97% identity) (49), it could be speculated that some ETEC strains have acquired supplementary genes for the full expression of colonization factors by horizontal gene transfer. Furthermore, VirulenceFinder found *csvA* (CS7) in 24 genomes of which 23 were also positive for *csfBCD* but negative for *csfA*. Considering that *csvA* (CS7) and *csfA* (CS5) are 91.1% identical, it could be speculated that *csvA* has replaced *csfA* in these strains. While examining such gene exchange in the tested 1,083 sequences is beyond the scope of this study, expanding the VirulenceFinder tool with these ETEC-associated gene alleles will hopefully encourage users to conduct closer analyses. The issue regarding overlapping genes and the differences in output using assembled genomes and/or reads also highlights the importance of detailed sequence analyses. In summary, our findings illustrate that it may be difficult to estimate the CF profiles due to multiple gene combinations because some are identical or near identical to each other. Finally, we observed a low level (13%) of human- and animal-specific CFs in the same genomes.

Apart from 30 non-ETEC genomes that could be categorized as either ExPEC_JJ_, UPEC_HM_, or both, this study identified four ETEC-ExPEC_JJ_ and one ETEC-UPEC_HM_ genomes. Several studies have identified hybrid STEC-ETEC strains from both humans and animals (1–7). EPEC expressing LT of ETEC has also been described (50). Therefore, it is important to include these ETEC-related genes in a more comprehensive VirulenceFinder tool at the CGE website in order to obtain more complete coverage of the virulence gene repertoire of pathogenic types of *E. coli*.

The standard nomenclature of *est* and *elt* genes is critical to minimize mistakes when analyzing these genes. Current nomenclature is insufficient to classify whether an isolate contains porcine or human-associated *est* genes. Additionally, it is impossible to distinguish between LT types based on the *elt* genes, as the gene name does not refer to the individual LT type. However, the new nomenclature meets these criteria, thus easing analyses.

We observed minor differences in the identification of genes when results obtained by assembled sequences were compared with those obtained by short-read FASTQ files. We would therefore recommend that users examine both kinds of sequence data with the VirulenceFinder web tool. Matches with an identity of 95% or higher are likely due to point mutations or poor sequencing. Depending on the length of the gene, an identity score of between 95% and 92% could represent new alleles. However, specific guidelines on how to define gene alleles, subtypes, and variants are generally problematic and invite international collaboration between dedicated researchers and experts.

### Future perspectives

ETEC vaccines are of great importance due to the severity of the infections, primarily in children. A tool such as this could assist in the surveillance of ETEC in order to determine the prevalence of relevant types in different parts of the world, allowing vaccine developers to target the most prevalent types and, thus, a more effective vaccine.

## Data Availability

The genomes analyzed here are part of BioProjects PRJEB33365, PRJNA421191, and PRJNA416134. Data Set S1 lists the accession numbers of all genomes included in the study. The VirulenceFinder database, which includes the ETECFinder database, can be downloaded from the CGE website (genomicepidemiology / virulencefinder_db — Bitbucket).

## SUPPLEMENTAL MATERIAL

Data Set S1 jcm.00570-23-s0001.xlsx Raw data.

Supplemental figures jcm.00570-23-s0002.docx Figures S1 to S3.

Supplemental tables jcm.00570-23-s0003.docx Tables S1 to S9.

ASM does not own the copyrights to Supplemental Material that may be linked to, or accessed through, an article. The authors have granted ASM a non-exclusive, world-wide license to publish the Supplemental Material files. Please contact the corresponding author directly for reuse.
