# Supplementary material for: Construction of the ETECFinder database for the characterization of enterotoxigenic Escherichia coli (ETEC) and revision of the VirulenceFinder web tool at the CGE website
Source: J Clin Microbiol. 2024 Apr 24;62(6):e00570-23. doi: 10.1128/jcm.00570-23 (PMC11237473; doi:10.1128/jcm.00570-23)
Supplement: Supplemental figures — Figures S1 to S3. [file jcm.00570-23-s0002.docx]

**Supplemental Figures 1 to 3.**

**Supplementary Figure 1**. Phylogroups represented in the dataset in which ETECFinder was tested on.

**Supplementary Figure 2**. A total of 168 different multi-locus sequence types (MLSTs) were predicted across all 1,083 *E. coli* genomes analysed. Here, the MLSTs with at least 5 genomes are shown.

**Supplementary Figure 3**. Bar graph showing the 25 most common countries the *E. coli* samples originate from.
