# Supplementary material for: Construction of the ETECFinder database for the characterization of enterotoxigenic Escherichia coli (ETEC) and revision of the VirulenceFinder web tool at the CGE website
Source: J Clin Microbiol. 2024 Apr 24;62(6):e00570-23. doi: 10.1128/jcm.00570-23 (PMC11237473; doi:10.1128/jcm.00570-23)
Supplement: Supplemental tables — Tables S1 to S9. [file jcm.00570-23-s0003.docx]

**Supplementary Table 1.** Alterations needed after revision of the *VirulenceFinder* database covering both colonization factors and enterotoxins.

| **Gene** | **Protein function used in the original *VirulenceFinder* database** | **No. of alleles in the original**  **database** | **New gene name** | **New name of the protein function in the revised database** | **No. of alleles in the revised database** |
| --- | --- | --- | --- | --- | --- |
| *cfa_c* | Colonisation factor antigen I | 4 1 | *cfaA cfaB cfaC cfaE*  *cfaD* | CFA/1 Major pilin  CFA/1 Major pilin chaperone CFA/1 Usher  CFA/1 Pilus length modulator CFA/1 Minor pilin | 2  4  2  1  4 |
| *cofA* | Longus type IV pilus subunit | 1 | *cofR cofS cofT cofA cofB cofC cofD cofE cofF cofG cofH cofI cofJ cofP* | CS8 Regulator  CS8 AraC-like protein CS8 Unknown  CS8 Type IV pilin precursor; major pilin CS8 Minor pilin  CS8 Unknown  CS8 Lipoprotein; secretin  CS8 Inner membrane accessory protein (IMAP) CS8 Inner membrane accessory protein (IMAP) CS8 Inner membrane accessory protein (IMAP) CS8 Nucleotide-binding protein; assembly ATPase  CS8 Integral membrane protein; inner membrane core protein (IMCP)  CS8 Secreted colonization factor CS8 Prepilin peptidase | 2  2  2  2  2  2  2  2  2  2  2  2  2  2 |
| *f17-A*  *f17-G* | Subunit A of F17 fimbrial protein  Adhesin subunit of F17 fimbriae | 7  9 | *F17A F17C F17D F17G* | Major fimbrial subunit of F17 F17 Usher  F17 Chaperone  Minor adhesive subunit of F17 fimbriae | 17  11  10  15 |
| *fanA* | Involved in biogenesis of F5 (K99) fimbriae | 1 | *fanA fanB fanC fanD fanE fanF*  *fanG fanH* | F5 (K99) Transcriptional regulator F5 (K99) Regulator  F5 (K99) Major fimbrial subunit F5 (K99) Usher  F5 (K99) Chaperone  F5 (K99) Minor fimbrial subunits F5 (K99) Minor subunit  F5 (K99) Minor subunit | 1  1  3  1  2  1  1  2 |

| *fasA* | Fimbriae 987P/F6 subunit | 1 | *fasA fasB fasC fasD fasE fasF fasG*  *fasH* | F6 (987P) Major subunit  F6 (987P) Major subunit chaperone F6 (987P) Adhesin-specific chaperone F6 (987P) Outer membrane usher  F6 (987P) Chaperone-like  F6 (987P) Minor subunit linker F6 (987P) Minor adhesive subunit  F6 (987P) Positive regulator, previously *fapR* (8) | 1  1  1  1  1  1  1  1 |
| --- | --- | --- | --- | --- | --- |
| *fedA fedF* | Fimbrial F107 subunit A Fimbrial adhesin AC precursor | 3  6 | *fedAab fedAac fedAnt fedB fedC*  *fedE fedF* | F18 (F107) Major serotype specific fimbrial subunit serotype ab F18 (F107) Major serotype specific fimbrial subunit serotype ac F18 (F107) Major serotype specific fimbrial subunit serotype nt F18 (F107) Usher  F18 (F107) Chaperone  F18 (F107) Minor fimbrial subunits F18 (F107) Minor adhesive subunit | 5  11  1  2  1  3  12 |
| *K88ab* | K88/F4 subunit ^2^ | 10 | *faeA faeB faeC faeD faeE faeF faeGab faeGac faeGad faeGW faeH faeI*  *faeJ* | F4 Regulator F4 Partial  F4 Minor fimbrial subunits F4 Usher  F4 Chaperone  F4 Minor fimbrial subunit  F4ab, Major serotype specific fimbrial subunit F4ac  F4ad F4W  F4 Fimbrial minor subunit F4 Fimbrial minor subunit F4 Fimbrial minor subunit | 2  2  4  8  9  8  3  14  1  1  8  17  5 |
| *fimF41* | Mature Fim41a/F41 protein | 2 | *fim41* | FimF41 Major fimbrial subunit | 4 |
| *lngA* | Longus type IV pilus | 2 | *lngX1 lngR lngS lngT lngX2 lngA lngB lngC lngD*  *lngE lngF* | -  CS21 Putative *papB*-like regulator CS21 Putative AraC-like regulator  -  -  CS21 Major pilin subunit CS21 Minor pilin subunit  -  CS21 Putative outer membrane protein  CS21 Putative inner membrane protein CS21 Putative inner membrane protein | 1  2  3  1  3  3  1  1  3  1  1 |

|  |  |  | *lngG lngH lngI lngJ lngP* | CS21 Putative periplasmic protein  CS21 Putative nucleotide-binding protein CS21 Putative inner membrane protein CS21 Putative ATPase-like  CS21 Putative prepilin peptidase | 2  2  2  3  1 |
| --- | --- | --- | --- | --- | --- |
| *ltcA* ^3^ | Heat-labile enterotoxin A subunit | 17 | *eltI*AB*_1-30*  *eltI*ABp | Heat-labile enterotoxin holotoxin AB subunit sequences; LTIh1-LTIh30 ^4^  LTIp | 31  1 |
| *sta1* | Heat-stabile enterotoxin ST-Ia | 2 | *estap*  *estah* | Heat-stable enterotoxin STa1, STa4, STa5, STa6, STa7, STa8 and STa9 porcine variants ^5^  Heat-stable enterotoxin STa2 and STa3 human variants | 15 ^6^  4 |
| *stb* | Heat-stabile enterotoxin II | 3 | *estb* | Heat-stable enterotoxin STb1 porcine variants Heat-stable enterotoxin STb2 human variant | 3  1 |

^1^ Two *cfaC* alleles in the original database were actually *csaA* (CS4) and *csuA* (CS14) respectively

^2^ Seven of these were actually F4ac, one F4ad and one F4W.

^3^ The original designation *ltcA* (“c” for chicken) was deleted. The revision included full holotoxin *eltIAB* sequences for 31 LTI types.

^4^ Three different nucleotide alleles had synonymous nucleotide substitutions: Acc. JX504011 = CP000795, EU113242 = EU113244, and EU113244 = EU113245.

^5^ STa7, STa8 and STa9 variants all have frameshifts, but they could be sequencing errors.

^6^ Two different STa4 alleles that had synonymous nucleotide substitutions: Acc. CP012501 = KT992796.

**Supplementary Table 2.** LT types based on the specific combination of the A and B subunit.

| **LT-Type** | **A subunit** | **B subunit(s)** |
| --- | --- | --- |
| LT1 LT1p | 1  1p, 4, 6 | 1, 10, 12, 13, 18, 21, 23, 24, 25, 26, 27, 28 |
| LT2 | 2 | 2, 7, 15, 16, 22 |
| LT3 | 3, 5 | 3, 8 |
| LT4 | 1p, 4, 6 | 4 |
| LT5 | 3, 5 | 5 |
| LT6 | 1p, 4, 6 | 6 |
| LT7 | 7 | 2, 7, 15, 16, 22 |
| LT8 | 8 | 3, 8 |
| LT9 | 9 | 9, 11, 19 |
| LT10 | 10 | 1, 10, 12, 13, 18, 21, 23, 24, 25, 26, 27, 28 |
| LT11 | 11 | 9, 11, 19 |
| LT12 | 12 | 1, 10, 12, 13, 18, 21, 23, 24, 25, 26, 27, 28 |
| LT13 | 13 | 1, 10, 12, 13, 18, 21, 23, 24, 25, 26, 27, 28 |
| LT14 | 14 | 14 |
| LT15 | 15 | 2, 7, 15, 16, 22 |
| LT16 | 16 | 2, 7, 15, 16, 22 |
| LT17 | 17 | 17, 20, 29, 30 |
| LT18 | 18 | 1, 10, 12, 13, 18, 21, 23, 24, 25, 26, 27, 28 |
| LT19 | 19 | 9, 11, 19 |
| LT20 | 20 | 17, 20, 29, 30 |
| LT21 | 21 | 1, 10, 12, 13, 18, 21, 23, 24, 25, 26, 27, 28 |
| LT22 | 22 | 2, 7, 15, 16, 22 |
| LT23 | 23 | 1, 10, 12, 13, 18, 21, 23, 24, 25, 26, 27, 28 |
| LT24 | 24 | 1, 10, 12, 13, 18, 21, 23, 24, 25, 26, 27, 28 |
| LT25 | 25 | 1, 10, 12, 13, 18, 21, 23, 24, 25, 26, 27, 28 |
| LT26 | 26 | 1, 10, 12, 13, 18, 21, 23, 24, 25, 26, 27, 28 |
| LT27 | 27 | 1, 10, 12, 13, 18, 21, 23, 24, 25, 26, 27, 28 |
| LT28 | 28 | 1, 10, 12, 13, 18, 21, 23, 24, 25, 26, 27, 28 |
| LT29 | 29, 30 | 17, 20, 29, 30 |
| LT30 | 29, 30 | 17, 20, 29, 30 |

**Supplementary Table 3.** ETEC associated genes including CF type or protein name, and number of alleles added as new to the *VirulenceFinder* database.

| **Gene** | **Description** | **CF type or name of protein** | **No. of alleles** |
| --- | --- | --- | --- |
| *cooA* | Major subunit | CS1 | 1 |
| *cooB* | Chaperone | CS1 | 1 |
| *cooD* | Chaperone | CS1 | 1 |
| *cooC* | Usher | CS1 | 1 |
| *cotB* | Major pilin chaperone | CS2 | 1 |
| *cotA* | Major pilin chaperone | CS2 | 1 |
| *cotC* | Usher | CS2 | 1 |
| *cotD* | Chaperone | CS2 | 1 |
| *cstA* | Major pilin chaperone | CS3 | 1 |
| *cstBCDEF* | Outer membrane usher | CS3 | 6 1 |
| *cstG* | Major pilin | CS3 | 3 |
| *cstH* | Major pilin reported (1) but not found | CS3 | - |
| *csaA* | Periplasmic chaperone-like protein | CS4 | 1 |
| *csaB* | CS4 major fimbriae subunit | CS4 | 1 |
| *csaC* | Usher protein | CS4 | 1 |
| *csaE* | Tip-associated protein | CS4 | 1 |
| *csaD*’ | Deleted regulatory protein | CS4 | 1 |
| *csfA* | major subunit | CS5 | 1 |
| *csfB* | coli surface factor five B, Chaperone | CS5 | 1 |
| *csfC* | Outer membrane usher protein | CS5 | 1 |
| *csfE* | Pilus length modulator | CS5 | 1 |
| *csfF* | Minor pilin chaperone | CS5 | 1 |
| *csfD* | Minor fimbrial subunit | CS5 | 1 |
| *cssA* | Structural subunit A | CS6 | 4 |
| *cssB* | Structural subunit B | CS6 | 2 |
| *cssC* | Periplasmic chaperone | CS6 | 8 |
| *cssD* | Usher protein | CS6 | 4 |
| *csvA* | Major subunit | CS7 | 1 |
| *cswA* | Major subunit | CS12 | 1 |
| *cswB* ^2^ | Chaperone | CS12 | 1 |
| *cswC* | Chaperone | CS12 | 1 |
| *cswD* | Outer membrane usher | CS12 | 1 |
| *cswE* | Chaperone | CS12 | 1 |
| *cswF* | Minor subunit | CS12 | 1 |
| *cswG* | Putative adhesin | CS12 | 1 |
| *cswR* | Transcriptional activator | CS12 | 1 |
| *cshA* | Minor pilin | CS13 | 1 |
| *cshB* | Usher | CS13 | 1 |
| *cshC* | Chaperone | CS13 | 1 |

| *cshD* | Minor pilin | CS13 | 1 |
| --- | --- | --- | --- |
| *cshE* | Pilin | CS13 | 1 |
| *cshF* | Minor pilin | CS13 | 1 |
| *cshG* | Minor pilin | CS13 | 1 |
| *csuA* | Major fimbrial subunit | CS14 | 3 |
| *csuB* | Periplasmic chaperone | CS14 | 1 |
| *csuC* | Outer membrane usher | CS14 | 1 |
| *csuD* | Minor fimbrial subunit | CS14 | 1 |
| *nfaA* | Nonfimbrial adhesin A | CS15 | 1 |
| *csbB* | Chaperone | CS17 | 2 |
| *csbA* | Fimbriae major subunit | CS17 | 1 |
| *csbC* | Usher | CS17 | 1 |
| *csbD* | Assembly protein/minor subunit | CS17 | 2 |
| *fotA* | Major subunit | CS18 | 2 |
| *fotB* | Major chaperone | CS18 | 1 |
| *fotC* | Minor chaperone | CS18 | 1 |
| *fotD* | Usher | CS18 | 1 |
| *fotE* | Minor chaperone | CS18 | 1 |
| *fotF* | Minor subunit | CS18 | 1 |
| *fotG* | Minor subunit-adhesin | CS18 | 1 |
| *fotS* | Site-specific recombinase | CS18 | 1 |
| *fotT* | Site-specific recombinase | CS18 | 1 |
| *csdB* | Periplasmic chaperone | CS19 | 1 |
| *csdA* | Major fimbrial subunit | CS19 | 1 |
| *csdC* | Outer membrane usher | CS19 | 1 |
| *csdD* | Minor fimbrial subunit | CS19 | 1 |
| *csnA* | Fimbria major subunit | CS20 | 2 |
| *csnB* ^2^ | Putative major subunit chaperone | CS20 | 2 |
| *csnC* | Putative adhesin-specific chaperone | CS20 | 2 |
| *csnD* | Putative outer membrane fimbrial usher | CS20 | 2 |
| *csnE* | Putative chaperone-like | CS20 | 1 |
| *csnF* | Putative minor subunit | CS20 | 2 |
| *csnG* | Putative minor adhesive tip subunit | CS20 | 2 |
| *cseA* | Adhesin protein | CS22 | 1 |
| *aalR* | Putative transcriptional regulator | CS23 | 1 |
| *aalA* | Minor structural subunit | CS23 | 1 |
| *aalB* | Usher | CS23 | 1 |
| *aalC* | Chaperone | CS23 | 1 |
| *aalD* | Minor structural subunit | CS23 | 1 |
| *aalE* | Major structural subunit | CS23 | 1 |
| *aalF* | Minor structural subunit | CS23 | 1 |
| *aalG* | Minor structural subunit | CS23 | 1 |

| *aalH* | Minor structural subunit | CS23 | 1 |
| --- | --- | --- | --- |
| *crsB* | Molecular chaperone | CS26 | 1 |
| *crsC* | Fimbria/pilus periplasmic chaperone | CS26 | 1 |
| *crsD* | Fimbrial biogenesis outer membrane usher protein | CS26 | 1 |
| *crsE* | Fimbria/pilus periplasmic chaperone | CS26 | 1 |
| *crsF* | Fimbrial protein | CS26 | 1 |
| *crsG* | Hypothetical protein | CS26 | 1 |
| *crsH* | Coli surface antigen | CS26 | 2 |
| *crsS* | Tyrosine-type DNA invertase | CS26 | 1 |
| *crsT* | Tyrosine-type DNA invertase | CS26 | 1 |
| *cmaHa* | Coli surface antigen a | CS27A | 1 |
| *cmaHb* | Coli surface antigen b | CS27B | 1 |
| *cnmHa* | Coli surface antigen a | CS28A | 1 |
| *cnmHb* | Coli surface antigen b | CS28B | 1 |
| *csmS* | Site specific recombinase | CS30 | 1 |
| *csmT* | Site specific recombinase | CS30 | 1 |
| *csmA* | Major subunit | CS30 | 1 |
| *csmB* | Chaperone | CS30 | 1 |
| *csmC* | Chaperone | CS30 | 1 |
| *csmD* | Usher | CS30 | 1 |
| *csmE* | Chaperone | CS30 | 1 |
| *csmF* | Minor subunit | CS30 | 1 |
| *csmG* | Fimbrial adhesin | CS30 | 1 |
| *cosA* | Major fimbrial subunit | PCFO71 | 1 |
| *cosB* | Periplasmic chaperone | PCFO71 | 1 |
| *cosC* | Outer membrane usher | PCFO71 | 1 |
| *cosD* | Minor fimbrial subunit | PCFO71 | 1 |
| *Tia* | Invasion determinant | Tia | 9 |
| *tibA* | Adhesin/invasin (Glycoprotein) | TibA | 4 |
| *tibC* | Glycosyltransferase | TibC | 7 |
| *etpA* | Invasin and adherence | EtpA | 19 |
| *etpB* | Nonfimbrial adhesin/TPS transporter | EtpB | 4 |
| *etpC* ^4^ | Glycotransferase | EtpC | 9 |
| *tleA* | *tsh*-like ETEC autotransporters/Glycosyltransferase | TleA | 2 |
| *yghJ* | Metalloprotease | YghJ | 5 |

^1^ *cstB* contains 11 ORFs of which five are considered to be functional usher genes *cstB* (2 alleles), *cstC*, *cstD*, *cstE*, and *cstF* (one each)

^2^ *cswB* (CS12) is identical to *csnB* (CS20)

^4^ EtpC (and EtpDEFGHIJKLMNO) has first been described by Makino *et al.* in 1998 in O157:H7 as a plasmid encoded type II secretion pathway related protein EtpC (2). EtpC has also been found in EPEC strains of serotype O103:H2 (3) and O55:H7 (4). EtpC in STEC or EPEC strains has no resemblance to the EtpC protein,

a putative nonfimbrial adhesin/TPS transporter/glycotransferase, and one of three genes in the ETEC two- partner secretion locus (*etpBAC*), found in ETEC strains (5).

1. **Madhavan TP, Sakellaris H**. 2015. Colonization factors of enterotoxigenic *Escherichia coli*. Adv Appl Microbiol **90**:155-97. 10.1016/bs.aambs.2014.09.003
2. **Makino K, Ishii K, Yasunaga T, Hattori M, Yokoyama K, Yutsudo CH, Kubota Y, Yamaichi Y, Iida T, Yamamoto K, Honda T, Han C-G, Ohtsubo E, Kasamatsu M, Hayashi T, Kuhara S, Shinagawa H**. Complete nucleotide sequences of 93-kb and 3.3-kb plasmids of an EHEC O157H7 derived from Sakai outbreak DNA Res **5**:1-9. https://doi.org/10.1093/dnares/5.1.1
3. **Ogura Y, Ooka T, Iguchi A, Toh H, Asadulghani M, Oshima K, Kodama T, Abe H, Nakayama K, Kurokawa K, Tobe T, Hattori M, Hayashi T**. 2009. Comparative genomics reveal the mechanism of the parallel evolution of O157 and non-O157 enterohemorrhagic *Escherichia coli*. Proc Natl Acad Sci U S A **106**:17939-17944. <http://www.ncbi.nlm.nih.gov/pubmed/19815525>
4. **Kyle JL, Cummings CA, Parker CT, Quinones B, Vatta P, Newton E, Huynh S, Swimley M, Degoricija L, Barker M, Fontanoz S, Nguyen K, Patel R, Fang R, Tebbs R, Petrauskene O, Furtado M, Mandrell RE**. 2012. *Escherichia coli* serotype O55:H7 diversity supports parallel acquisition of bacteriophage at Shiga toxin phage insertion sites during evolution of the O157:H7 lineage. J Bacteriol **194**:1885-1896. JB.00120-12 [pii];10.1128/JB.00120-12 [doi]
5. **Fleckenstein JM, Roy K, Fischer JF, Burkitt M**. 2006. Identification of a two-partner secretion locus of enterotoxigenic *Escherichia coli*. Infect Immun **74**:2245-58. 10.1128/IAI.74.4.2245-2258.2006

**Supplementary Table 4.** Description of enterotoxins and the alleles as well as their gene names. Changes to the current database are indicated.

| **LT-type** | **ST-type** | **SUM** |
| --- | --- | --- |
| **LT1b** | *estap*; STap1-01 | 14 |
| **LT3** | - | 6 |
| **LT7** | - | 2 |
| **LT8** | - | 14 |
| **LT11** | - | 10 |
| **LT12** | - | 2 |
| **LT12+LT13+LT25** | - | 1 |
| **LT12b** | - | 2 |
| **LT13** | - | 17 |
| **LT15** | *estah*; STah3-01 | 39 |
| **LT15** | *estah;* STah2-01 | 19 |
| **LT15** | *estah;* STah3-05 | 3 |
| **LT15** | - | 23 |
| **LT15b** | - | 11 |
| **LT17** | - | 25 |
| **LT18** | *estap*; STap1-02 | 21 |
| **LT18** | *estap*; STap1-02; *estah*; STah3-02 | 8 |
| **LT18** | *estap*; STap1-02b | 1 |
| **LT18** | - | 2 |
| **LT18b** | *estap*; STap1-02 | 6 |
| **LT18c** | *estap*; STap1-01 | 1 |
| **LT19** | *estap*; STap1-01 | 1 |
| **LT20** | *estah*; STah3-01 | 3 |
| **LT21** | - | 1 |
| **LT22** | - | 1 |
| **LT23** | *estap*; STap1-01 | 12 |
| **LT24** | *estap*; STap1-01 | 12 |
| **LT25** | *estap*; STap1-01 | 6 |
| **LT26** | *estap*; STa7-01 | 1 |
| **LT27** | *estap*; STap1-01 | 1 |
| **LT28** | *estap*; STap1-01 | 1 |
| **LT29** | *estah*; STah3-01 | 3 |
| **LT30** | *estah*; STah3-01 | 90 |
| **LT30** | *estap*; STap1-01 | 1 |
| **New LT31** | *estap*; STap1-01 | 1 |
| **New LT32** | *estap*; STap1-01 | 1 |
| **New LT32** | *estap*; STap1-05 | 14 |
| **New LT32** | - | 51 |
| **New LT33** | *estap*; STap1-01 | 15 |
| New LT33 | - | 12 |
| **LTp1 (previous LT4)** | *estap*; STap1-01, *estb*; STb1-01 | 13 |
| **LTp1 (previous LT4)** | *estap*; STap1-01, *estb*; STb1-03 | 4 |
| **LTp1 (previous LT4)** | *estb*; STb1-01 | 107 |
| **LTp1 (previous LT4)** | *estb*; STb1-01-NEW | 1 |

| **LTp1 (previous LT4)** | - | 3 |
| --- | --- | --- |
| - | *estah*; STah3-01 | 88 |
| - | *estah*; STah3-02 | 38 |
| - | *estah;* STah2-01 | 63 |
| - | *estah;* STah2-02 | 1 |
| - | *estah;* STah3-04 | 1 |
| - | *estah;* STah3-06 | 2 |
| - | *estap*; STap1, *estb*; STb1-03 | 1 |
| - | *estap*; STap1-01 | 17 |
| - | *estap*; STap1-01, *estb*; STb1-01 | 1 |
| - | *estap*; STap1-01, *estb*; STb1-03 | 11 |
| - | *estap*; STap1-03 | 78 |
| - | *estap*; STap1-06 | 1 |
| - | *estap*; STap4-04 | 2 |
| - | *estap*; STap5-07 | 1 |
| - | *estb*; STb1-01 | 1 |
| - | - | 193 |
| **Total** |  | **1083** |

**Supplementary Table 5.** Combinations of CF genes found in 441 ETEC and 11 non-ETEC sequences. Presence of one gene specific for a CF is enough to be reported, *i.e*., all genes encoding a CF may not be present in all CF combinations. The specific allele combination findings are listed below this table.

| **Combinations** | **ETEC** | **Non-ETEC** | **Total** |
| --- | --- | --- | --- |
| CFA/I; CS1 |  | 2 | 2 |
| CFA/I; CS1; CS17; CS21 |  | 1 | 1 |
| CFA/I; CS1; CS21 |  | 2 | 2 |
| CFA/I; CS1; CS3 | 1 |  | 1 |
| CFA/I; CS1; CS3; CS17; CS21 | 21 |  | 21 |
| CFA/I; CS1; CS3; CS21 | 15 |  | 15 |
| CFA/I; CS12 | 1 |  | 1 |
| CFA/I; CS12; CS21 | 12 |  | 12 |
| CFA/I; CS14 | 43 |  | 43 |
| CFA/I; CS2; CS21 | 1 |  | 1 |
| CFA/I; CS2; CS3 | 2 |  | 2 |
| CFA/I; CS2; CS3; CS21 | 30 |  | 30 |
| CFA/I; CS2; CS3; CS21; CS23 | 1 |  | 1 |
| CFA/I; CS21 | 77 |  | 77 |
| CFA/I; CS3 | 6 |  | 6 |
| CFA/I; CS3; CS21 | 19 |  | 19 |
| CFA/I; CS4; CS21 | 2 | 1 | 3 |
| CFA/I; CS4; CS6 | 12 |  | 12 |
| CFA/I; CS6 | 75 | 2 | 77 |
| CFA/I; CS6; CS23 | 3 |  | 3 |
| CFA/I; CS6; CS8 | 6 |  | 6 |
| CS12; CS13 | 6 |  | 6 |
| CS12; CS13; CS26 |  | 2 | 2 |
| CS12; CS13; CS30 | 2 |  | 2 |
| CS12; CS20 | 21 |  | 21 |
| CS12; CS20; CS23 | 1 |  | 1 |
| CS12; CS23 | 3 |  | 3 |
| CS12; CS23; CS26 | 3 |  | 3 |
| CS12; CS26 | 14 |  | 14 |
| CS18; CS30 | 13 |  | 13 |
| CS2; CS21 |  | 1 | 1 |
| CS2; CS3; CS21 | 1 |  | 1 |
| CS23; F17 | 1 |  | 1 |
| CS4; CS6 | 2 |  | 2 |
| CS5; CS6 | 55 |  | 55 |
| CS5; CS6; CS7 | 1 |  | 1 |
| CS5; CS7 | 23 |  | 23 |
| CS6; CS12 | 18 |  | 18 |
| CS6; CS12; CS13 | 1 |  | 1 |
| CS6; CS12; CS18 | 1 |  | 1 |
| CS6; CS21 | 39 |  | 39 |
| CS6; CS8 | 8 |  | 8 |
| CS8; F17 | 1 |  | 1 |
| **Total:** | **541** | **11** | **556** |

# Specific allele combination findings

The full gene cluster (*cfaA*, *cfaB*, *cfaC*, *cfaD* and *cfaE* genes) encoding CFA/I was found in 81 genomes, but in addition to these, 79 genomes had two different *cfaD* genes, all of which were found on two different contigs at identities ranging from 93 % to 100 %. *cfaD* was found once as the only CFA/I gene in 217/338 genomes. *cfaB* and *cfaD* were found in 33/338 genomes. The individual genes *cfaA*, *cfaB*, *cfaC*, *cfaD* and *cfaE* were found in 82, 121, 82, 410 and 82 genomes, respectively. CS1 (*cooABCD*) was found in four genomes and *cooACD* in 38 genomes. CS2 genes were complete (*cotABCD*) in 36 sequences. CS3 genes were found in 97 sequences, *cstA* (38), *cstB* (62), *cstC* (1), *cstD* (0), *cstE* (0), *cstF* (32), and *cstG* (96 once and one twice). CS4 genes were found in 18 sequences, *csaABCE* (13) and *csaD* (17 once and one twice). CS5 (*csfABCDEF*) genes were complete in 54 sequences. Individual genes were: *csfA* (55), *csfB* (78), *csfC* (77), *csfD* (78 once and one twice), *csfE* (79), and *csfF* (55). In 79 of these genomes, 24 were negative for *csfF* but positive for the *csvA* gene for CS7. *csvA* was also found in one genome positive for *cssABD* (CS6). CS6 (*cssABCD*) genes were complete in 90/240 sequences, *cssA* (237 once and one twice), *cssB* (238 once and one twice), *cssC* (151 once and one twice) and *cssD* (178). CS8 (*cofABCDEFGHIJPRST*) genes were complete in 8/22 sequences, *cofA* (8), *cofBCDEFGHIJPT* (19), and *cofRS* (23). CS12 (*cswABCDEFGR*) genes were complete in 27/142 sequences, and *cswR* the only gene in 115 genomes. CS13 (*cshABCDEFG*) genes were complete in 11 sequences. CS14 (*csuAA1A2BCD*) genes were complete in 25/44 sequences, *csuA* (16 once, 25 twice, one thrice and one four times), *csuA1* (27 once and twice in two), *csuA2* (26 once and twice in one), *csuBD* (44) and *csuC* (42). CS17 (*csbABCD*) genes were complete in 27/49 sequences, *csbABC* (27), and *csbD* (49). CS18 (*fotABCDEFGST*) genes were complete in 2/15 genomes with *fotT* as the only gene in 13 genomes. CS19 (*csdABCD*) genes were complete in 17 genomes. CS20 (*csnABCDEFG*) genes were complete in 11/27 sequences, *csnA* missing in the remaining 16 genomes. CS21 (*lngABCDEFGHIJPRSTX1X2*) genes were complete in 92/255 sequences. *lngBCEFGHJPRSTX2* (254; *lngX2* twice in one sequence), *lngD* (259), *lngI*

(262) and *lngX1* (253). The *cseA* gene for CS22 was found in two sequences. CS23 genes were found in 25 sequences, *aalA* (11), *aalB* (0), *aalCF* (12), *aalD* (2), *aalE* (1), *aalG* (9), *aalH* (18) and *aalR* (seven once, three twice and five thrice). CS26 genes were found in 19 sequences, *crsBHST* (5), *crsCFG* (19), *crsD* (1) and *crsE* (18). CS30 (*csmABCDEFGST*) genes were complete in 32 genomes. F17 genes were found in 147 sequences, *F17A* (8), *F17C* (10), *F17D* (141) and *F17G* (14). Add to this that using reads could give different results.

**Supplementary Table 6.** Single CF genes found in 174 ETEC, one ETEC-UPECHM, four ETEC-ExPECJJ, five ExPECJJ/UPECHM, one ExPECJJ, and 19 ETEC-ExPECJJ/UPECHM-negative sequences.

| **CF type single** | **ExPECJ**  **J** | **ExPECJJ**  **/UPECHM** | **negative** | **ETEC/**  **ExPECJJ** | **ETEC** | **ETEC/UPECH**  **M** | **SUM** |
| --- | --- | --- | --- | --- | --- | --- | --- |
| F17 |  |  | 8 |  | 21 | 1 | 30 |
| CS12 |  |  | 1 |  | 56 |  | 57 |
| CS21 |  |  | 3 |  | 29 |  | 32 |
| CS17 |  |  |  |  | 27 |  | 27 |
| CS30 |  |  |  |  | 17 |  | 17 |
| CS6 |  |  | 3 | 4 | 10 |  | 17 |
| CS23 | 1 |  |  |  |  |  | 1 |
| CS8 |  |  | 1 |  | 4 |  | 5 |
| CS20 |  | 5 |  |  |  |  | 5 |
| PCFO71 |  |  |  |  | 3 |  | 3 |
| CFAI ^1)^ |  |  |  |  | 3 |  | 3 |
| CS22 |  |  | 2 |  |  |  | 2 |
| CS14 |  |  |  |  | 1 |  | 1 |
| CS4 |  |  |  |  | 1 |  | 1 |
| CS18 |  |  |  |  | 1 |  | 1 |
| CS3 |  |  | 1 |  |  |  | 1 |
| **Total** | **1** | **5** | **19** | **4** | **174** | **1** | **203** |

^1)^ Found in serotypes O128ac:H45 (2 genomes) and O153:H46 (1).

# Specific single allele findings

The full gene cluster for F17 was not found with F17A (8), F17C (6 and 2 twice), F17G (5 and 3 thrice). F17D was found in 21 ETEC genomes and once in an ETEC/UPECHM genome. CS 12 (*cswABCDEFGR*) genes were complete in 25/57 genomes. *cswR* was the only gene found in the remaining 32 genomes. All CS21 (*lngBEFGHIJPRSTX2*) genes except *lngA* (1) and *lngX1* (31) were found in 32 genomes. CS17 (*csbABCD*) was complete in 27 genomes. CS30 (*csmABCDEFGST*) genes was complete in 17 genomes. CS6 genes *cssA* (16), *cssB* (17), *cssC* (9) and *cssD* (6) was found in 17 genomes. C23 (*aalACDEFGH* (1) and *aalR* (twice) were found in the same genome, and *aalACDHFR* (1), *aalCF* (1), *aalCH* (1), *aalH* (7) in nine different genomes. Except for *cofA* (0), CS8 genes (*cofBCDEFGHIJPRST*) were found in five genomes with *cofR* (twice). CS20 (*csnBCDEFG*) genes were found in five negative genomes, and *csnA* (0). PCFO71 (*cosABCD*) genes were complete in three sequences. CFAI genes *cfaABCE* (3) and *cfaD* (twice) were detected in three genomes. CS22 (*cseA*) was found in two negative genomes. *csaABCDE* (CS4), *csuBD* (CS14) and *fotABCDFGS* (CS18) were found once in three different genomes. *cstG* (CS3) was found twice in one genome.

**Supplementary Table 7.** Animal fimbriae alone or in combination with human CFAs found in 167 sequences.

| **F type** | **Human CFA** | **number** |
| --- | --- | --- |
| F4 |  | 11 |
| F4ac |  | 1 |
| F4 | CS23 | 6 |
| F4 | CS12; CS20; CS23 | 1 |
| F4 | CS12; CS23 | 3 |
| F4 | CS12; CS23; CS26 | 3 |
| F4; F17 | CS23 | 1 |
| F4; F17 |  | 3 |
| F4ab; F17 |  | 6 2) |
| F4ac; F17 |  | 73 |
| F4ad; F17 |  | 2 |
| F4; *fimF41* |  | 1 |
| F4; F5; *fimF41* | CS23 | 6 |
| F4; F6; F17 |  | 2 2) |
| F4ac; F6; F17 |  | 10 |
| F6 |  | 2 |
| F6 | CS8 | 2 |
| F6; F17 | CS8 | 1 |
| F6; F18ac; F17 |  | 1 2) |
| F18ab |  | 7 3) |
| F18ac |  | 7 1) |
| F18ac; F17 |  | 18 4) |
| Total |  | 167 |

^1)^ All were positive for *stx2e*-O139-S1191 (Acc. No. M21534)

^2)^ One was positive for *stx2e*-O139-S1191 (Acc. No. M21534)

^3)^ Six were positive for *stx2e*-O139-S1191 (Acc. No. M21534)

^4)^ Four were positive for *stx2e*-O139-S1191 (Acc. No. M21534).

Nine ETEC-ExPECJJ/UPECHM-negative sequences were positive for F4.

Twelve ETEC-ExPECJJ/UPECHM-negative sequences were positive for F18 of which nine were positive for

*stx2e*-O139-S1191 (Acc. No. M21534).

Three ExPECJJ/UPECHM sequences positive for F4, F4-CS23 and F4-*fimF41* respectively One ExPECJJ for F4.

One UPECHM was positive for F4-CS23.

**Supplementary Table 8.** Comparison of CF results from Hazen *et al.* (1) and the results using the revised VirulenceFinder on 269 genomes.

| **Colonization Factors** | **CF results by VirulenceFinder** | **difference** | **OK** | **OK++** | **Sum** |
| --- | --- | --- | --- | --- | --- |
| **CFA/I** | CFAI |  | 4 |  | 4 |
|  | CFAI; CS4 (*csaA*) |  |  | 1 | 1 |
|  | CFAI; CS5 (*csfA*) |  |  | 1 | 1 |
|  | CFAI; CS21 (*lngHIJP*) |  |  | 1 | 1 |
| **CFA/I*** | neg |  | 7 |  | 7 |
|  | CS22 (*cseA*) |  |  | 1 | 1 |
|  | CS21 (*lngHIJP*) |  |  | 1 | 1 |
| **CFA/I*, CS14** | CFAI (*cfaD*); CS14 |  |  | 2 | 2 |
| **CFA/I*, CS2, CS3** | CFAI (*cfaD*); CS2; CS3 |  |  | 1 | 1 |
| **CFA/I*, CS21** | CS21 |  | 5 |  | 5 |
|  | CFAI (*cfaD*); CS12 (*cswR*);  CS21 |  |  | 1 | 1 |
| **CFA/I*, CS22** | CS22 |  | 1 |  | 1 |
| **CFA/I, CS14, CS21** | CFAI; CS4 (*csaD*); CS14; CS21 |  |  | 1 | 1 |
| **CFA/I, CS21** | CFAI; CS21 |  | 124 |  | 124 |
|  | CFAI; CS4 (*csaD*); CS21 |  |  | 11 | 11 |
| **CS6** | CS6 |  | 2 |  | 2 |
| **CS6*** | neg |  | 13 |  | 13 |
|  | CFAI (*cfaD*); CS12 (*cswR*) |  |  | 1 | 1 |
|  | CS4 (*csaD*) |  |  | 1 | 1 |
| **CS6*, CFA/I, CS21** | CFAI; CS21 |  | 1 |  | 1 |
| **CS6*, CS14** | CFAI (*cfaD*); CS14 |  |  | 2 | 2 |
| **CS6, CS21** | CS6; CS21 |  | 24 |  | 24 |
|  | CFAI (*cfaD*); CS6 | 1 |  |  | 1 |
| **CS6, CS4, CS21** | CFAI (*cfaD*); CS4; CS6 | 5 |  |  | 5 |
| **CS6, CS5** | CS5; CS6 |  | 56 |  | 56 |
|  | CS5; CS6; CS21 (*lngP*) |  |  | 1 | 1 |
| **Total** |  | **6** | **237** | **26** | **269** |

* An asterisk indicates that the CF genes were identified by PCR in Hazen *et al.* (1). OK indicates accordance between LS-BSR and VirulenceFinder results.

OK++ indicates additional genes found by VirulenceFinder.

CF profiles determined by VirulenceFinder matched with LS-BSR in 237 genomes: CFAI (defined as positive for four out of five *cfa* genes) + CS21 (defined as positive for 14 out of 16 *lng* genes) was found in 125 genomes, CS5 (five out of six *csf* genes; *csfE* was not detected in any of the genomes) + CS6 (three out of four *css* genes) was found in 56 genomes, CS6+ CS21 (14 out of 16 *lng* genes) was found in 24 genomes, CF- negative (20), CS21 (5), CFAI (4), CS6 (2), and CS22 (1) were also found by VirulenceFinder. In 130 genomes, two copies of *cfaD* (CFAI) was found. In PNTM01000001.1 the full CFAI encoding cluster (*cfaABCDE*) was found on three contigs (PNTM01000137.1, PNTM01000154.1, PNTM01000058.1).

Additional CF genes that were not identified by LS-BSR or mentioned in Hazen *et al*. (1) were found in 26 genomes and included single genes *cfaD* (7), *csaD* (14), *csfA* (1), *lngHIJP* (2), *lngP* (1) and *cswR* (2). In six genomes *cfaD* was found by VirulenceFinder instead of *lng genes for* C21.

Specifically, in 11 sequences *csaD* from CS4 was also found in addition the CFAI and CS21 found by LS-BSR. Six sequences reported as negative for CFAI by LS-BSR were *cfaD* (CFAI) positive. Two of these were also positive for *cswR* (CS12), and two were positive for *lngHIJP* (CS21) and *cseA* (CS22) respectively with VirulenceFinder. Additional differences included genes found by VirulenceFinder for CFAI+ *csaA* (CS4), CFAI+*csfA* (CS5), CFAI+*lngHIJP* (CS21) [Three only CFA/I positive by LS-BSR]. *cfaD* (CFAI)+*cswR* (CS12) and *csaD* (CS4) were found in two CS6 LS-BSR negative. *cfaD* (CFAI)+CS14 was found twice in CS6 negative and CS14 positive by LS-BSR. *cfaD* (CFAI)+CS6 was found by VirulenceFinder in CS6+CS21 positive by LS-

BSR. In five CS4+CS6+CS21 positive by LS-BSR VirulenceFinder did not find *lng* genes for CS21, but *cfaD*

(CFAI)+CS4+CS6.

1. **Hazen TH, Nagaraj S, Sen S, Permala-Booth J, Del Canto F, Vidal R, Barry EM, Bitoun JP, Chen WH, Tennant SM, Rasko DA**. 2019. Genome and Functional Characterization of Colonization Factor Antigen I- and CS6-Encoding Heat-Stable Enterotoxin-Only Enterotoxigenic *Escherichia coli* Reveals Lineage and Geographic Variation. mSystems **4**. 10.1128/mSystems.00329-18

**Supplementary Table 9.** Additional gene alleles found after using the revised CGE webtool allowing for a 30 nucleotide overlap. *tibC o*verlaps with *tibA* by 8 nucleotides.

| **Colonization factor** | **gene** | **No.** | **gene** | **No.** | **gene** | **No.** | **gene** | **No.** | **Total** |
| --- | --- | --- | --- | --- | --- | --- | --- | --- | --- |
| CS8 | *cofE* | 10 | *cofG* | 9 |  |  |  |  | 19 |
| CS1 | *cooD* | 42 |  |  |  |  |  |  | 42 |
| PCFO71 | *cosD* | 3 |  |  |  |  |  |  | 3 |
| CS26 | *crsD* | 18 | *crsE* | 1 |  |  |  |  | 19 |
| CS17 | *csbC* | 1 | *csbD* | 48 |  |  |  |  | 49 |
| CS19 | *csdC* | 3 | *csdD* | 14 |  |  |  |  | 17 |
| CS5 | *csfE* | 76 |  |  |  |  |  |  | 76 |
| CS13 | *cshB* | 9 | *cshC* | 2 |  |  |  |  | 11 |
| CS30 | *csmB* | 1 | *csmC* | 31 | *csmD* | 20 | *csmE* | 12 | 64 |
| CS20 | *csnB* | 9 | *csnC* | 18 | *csnD* | 14 | *csnE* | 13 | 54 |
| CS6 | *cssC* | 67 | *cssD* | 25 |  |  |  |  | 92 |
| CS12 | *cswD* | 18 | *cswE* | 9 |  |  |  |  | 27 |
| CS18 | *fotD* | 1 | *fotE* | 1 |  |  |  |  | 2 |
| CS21 | *lngC* | 228 | *lngD* | 31 |  |  |  |  | 259 |
| F4 | *faeB* | 81 | *faeC* | 1 | *faeD* | 2 | *faeE* | 43 | 127 |
| F5 | *fanD* | 1 | *fanE* | 6 | *fanF* | 2 | *fanG* | 6 | 15 |
| F6 | *fasE* | 16 |  |  |  |  |  |  | 16 |
|  | *tibA* | 66 |  |  |  |  |  |  | 66 |
|  | *tibC* | 47 |  |  |  |  |  |  | 47 |
|  | *traT* | 46 |  |  |  |  |  |  | 46 |
| **Total:** |  | **743** |  | **196** |  | **38** |  | **74** | **1051** |
